# The Identity of the Cells in Fluid from Cystic Tumors of the Neck, Scrotum, Etc., with the So-called Ovarian Corpuscle, or Drysdale Cell

**Published:** 1879-07

**Authors:** Hugh M. Taylor

**Affiliations:** Assistant Demonstrator of Anatomy, Medical College of Virginia, etc., Richmond, Va.


### The Identity of the Cells found in Fluid from Cystic Tumors of the Neck, Scrotum, etc., with the so-called Ovarian Corpuscle or Drysdale Cell.

By Hugh M. Taylor, Assistant Demonstrator of Anatomy, Medical College of Virginia, etc., Richmond, Va.

Some cases have come under my notice recently which are, I think, just at this time interesting. A few weeks ago, Prof. Christopher Johnston, of Baltimore, Md., reported in the *Virginia Medical Monthly*,* that he had found in some fluid taken from a cystic tumor of the neck, cells very much resembling the Drysdale cell or the so-called ovarian cell. Shortly after reading this report, I was called upon to remove a tumor from the neck of a colored woman, which proved to be fibro-cystic in character. Wishing to ascertain if it contained any cells or corpuscles which resemble those usually found in the ovarian fluid, I sent some of it to Mr. Hugh Blair, of this city, accompanied with the request that he would submit it to a microscopic examination. Mr. Blair reported that there was a very striking resemblance between the cells he found in the fluid I gave him, and those usually found in ovarian dropsy.

A few days ago, I assisted Prof. Hunter McGuire to remove a testicle which had undergone cystic degeneration. I also obtained and sent a specimen of this fluid to Mr. Blair, and requested him to examine it. He reports that the resemblance between the cells found in the fluid from the cystic diseased testicle, to those found

* April, 1879, *Baltimore Academy of Medicine Proceedings.*

in ovarian dropsy, is even more striking than they were in the first specimen I gave him; and that if there are any characters sufficiently distinct to differentiate them from the Drysdale cell, he is, at this time, unable to point them out. * * * *

In starting this investigation, Prof. Johnston will, I am sure, add much to his already enviable reputation by refuting the idea which has so long prevailed, viz.: that these cells are alone to be found in cystic degeneration of the ovaries, and are therefore pathognomonic of ovarian dropsy. It may be shown that they are only to be found in a peculiar cystic degeneration of special glands. I think this hypothesis highly probable, since we have only found them in the tumors about the neck where glands are abundant, and in the ovary and testicle—analogous organs; but I hardly think they will again be claimed as being peculiar to ovarian dropsy.—*Virginia Medical Monthly.*

